# Supplementary material for: Analyses of Xenorhabdus griffiniae genomes reveal two distinct sub-species that display intra-species variation due to prophages
Source: BMC Genomics. 2024 Nov 15;25:1087. doi: 10.1186/s12864-024-10858-2 (PMC11566119; doi:10.1186/s12864-024-10858-2)
Supplement: Supplementary file 3 — Additional file 3. Word document with additional details of the detection of Xenorhabdus griffiniae defense systems and analysis of CRISPR loci. Table S4. Summary of defense systems in X. griffiniae and related strains. Table S5. Locus tags and coordinates of CRISPR-Cas features. Table S6. CRISPR spacers, protospacers, concomitant annotation and genome wherein they are located. Methods and results for X. griffiniae CRISPR repeats, protospacers and self-targeting immunity. [file 12864_2024_10858_MOESM3_ESM.docx]

**Analyses of *Xenorhabdus griffiniae* genomes reveal two distinct sub-species that display intra-species variation due to prophages**

Jennifer K. Heppert^1*^, Ryan Musumba Awori^2*^, Mengyi Cao^3^, Grischa Chen^3^, Jemma McLeish^1^, Heidi Goodrich-Blair^1#^

**Additional file 3. Word document with additional details of *Xenorhabdus griffiniae* defense system detection and analysis of CRISPR loci**

**Table S4. Summary of defense systems in *X. griffiniae* and related strains^a^**

| **Type^b^** | **Subtype^c^** | **HGB2511** | **ID10** | **Kalro** | **TH1** | **BMMCB** |
| --- | --- | --- | --- | --- | --- | --- |
| Abi | PD-T4-5 | X |  |  |  |  |
| Unknown | RloC | X |  |  |  |  |
| RM | RM_Type_III | X |  |  |  |  |
| Abi | Thoeris_I |  | X |  |  |  |
| Abi | AbiD |  |  | X |  |  |
| TA | PsyrTA |  |  | X |  |  |
| TA | ShosTA |  |  | X |  |  |
| Abi | Lamassu-Cap4_nuclease | XXX | XX |  |  |  |
| Unknown | PD-Lambda-5 | X | X |  |  |  |
| Retron | Retron_II | X | X |  |  |  |
| TA | AbiE | X | X | X |  |  |
| Plasmid defense | Wadjet_I | X | X | X |  |  |
| CAS | CAS_Class1-Subtype-I-E | XX | X | X | XX | X |
| TA | MazEF | X | X | X | X | X |
| TA | SanaTA | X |  | X | X |  |
| TA | PfiAT |  | X | X | X |  |
| RM | RM_Type_II | X | X |  |  | X |
| RM | RM_Type_I |  | X |  | X | X |
| Unknown | GAPS2 | X |  |  | X |  |
| Abi | CBASS_I |  | X |  | X |  |
| Possibly Abi | BstA |  |  | X | X |  |
| Unknown | Gao_Upx/Gao_Ppl |  |  | X | X |  |
| RM | RM_Type_IIG |  |  | X |  | X |
| Abi | PrrC |  |  |  | X | X |
| Unknown | Rst_3HP |  |  |  | X |  |
| Non-abortive phage defense | Shedu |  |  |  | X |  |

^a^Defense systems identified and classified by <https://defensefinder.mdmlab.fr> (accessed 6/28/2024). See Supplemental Sheet S22 in Additional file 2 for full list and details. Each "X" indicates a predicted copy of the indicated defense type. Orange shading indicates pathways that were identified only in *X. griffiniae* genomes. Blue shading indicates pathways that were identified in all genomes.

^b^The type of system was manually annotated based on Georjon, H., Bernheim, A. The highly diverse antiphage defence systems of bacteria. *Nat Rev Microbiol* **21**, 686–700 (2023). <https://doi.org/10.1038/s41579-023-00934-x> and <https://defensefinder.mdmlab.fr/wiki/>

RM: restriction-modification; Abi: abortive infection; TA: toxin/antitoxin system

^c^Subtype as determined by defensefinder; See Supplemental Sheet S22 in Additional file 2.

**Table S5**. Locus tags and coordinates of CRISPR-Cas features. The MaGe locus tags are listed for genes encoded at *cas* loci (region 1 for all genomes, and region 2 for HGB2511, ID10, and TH1) in each of the analyzed strains. Annotated gene names are provided on the left (for region 1) and on the right (for region 2) of the table. The genome start coordinate of the start codon for the *cas3* gene is provided at the bottom of the *cas* list. For each CRISPR repeat region found in a genome, the start coordinate of the first repeat and the number of spacers identified are provided.


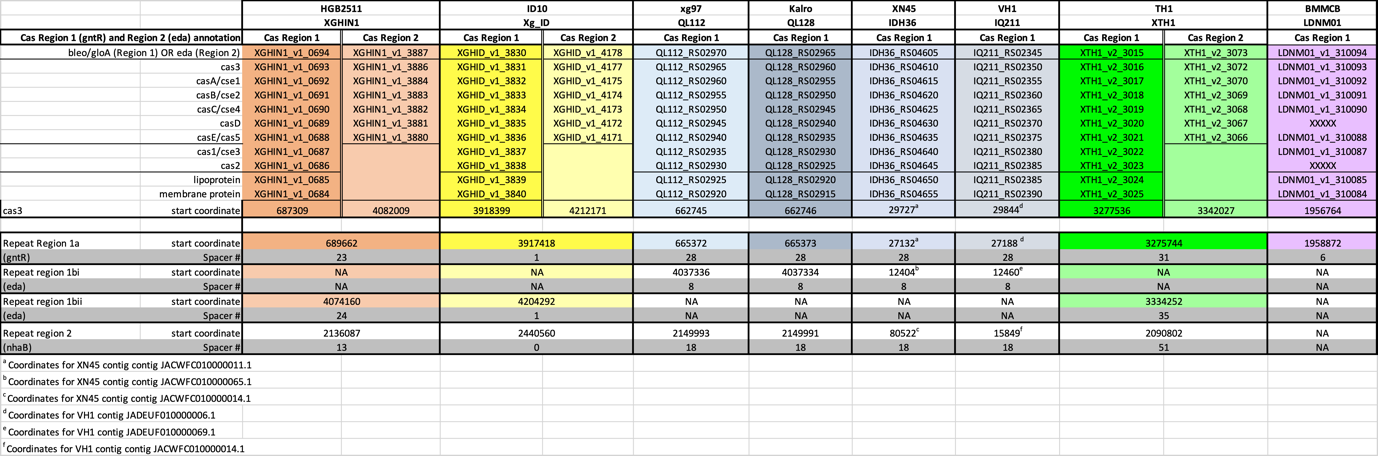


**Table S6**. Representative putative protospacers of *Xenorhabdus* strain CRISPR spacers, identified using sequence similarity searches in the CRISPRTarget platform (See Materials and Methods in main text). Shown are source strain of CRISPR spacer (Strain) and CRISPR spacer label (Spacer; See Fig. 8D-F and Table S4), and the annotation (Protospacer ID) and general category of annotated function (Protospacer Type) of each protospacer-containing locus. The blue highlighting indicates those spacers that also had protospacers among the closest relatives of *X. griffiniae*. The gold, green, and gray highlighting indicates protospacers from eukaryotic viruses, plasmids, or genes of unknown function, respectively. All other representative protospacers shown were in putative bacterial prophage genes.


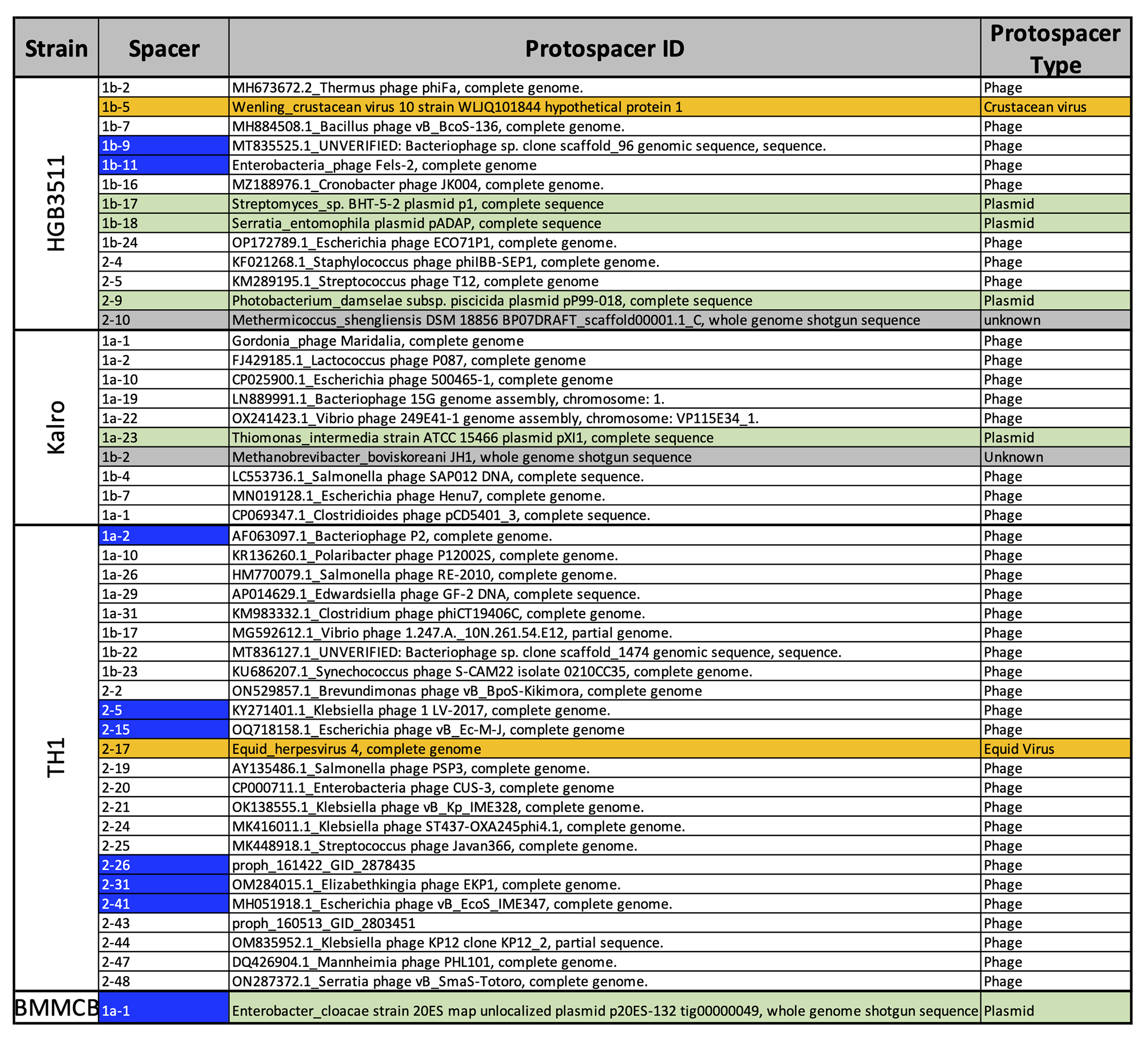


**Additional Information on CRISPR array identification in *X. griffiniae* and related strains**

HGB2511, Kalro, ID10, TH1, and BMMCB genomes were submitted to CRISPRdetect (<http://crispr.otago.ac.nz/CRISPRDetect/> CRISPRDetect 2.3) to identify potential CRISPR regions. The program did not detect any repeats in ID10. The consensus repeat sequences (29 bp) for each of the identified regions (here referred to as Region 1a, Region 1b, and Region 2 – see main text) of the other strains is shown below. Variability within the region among strains is highlighted in green.


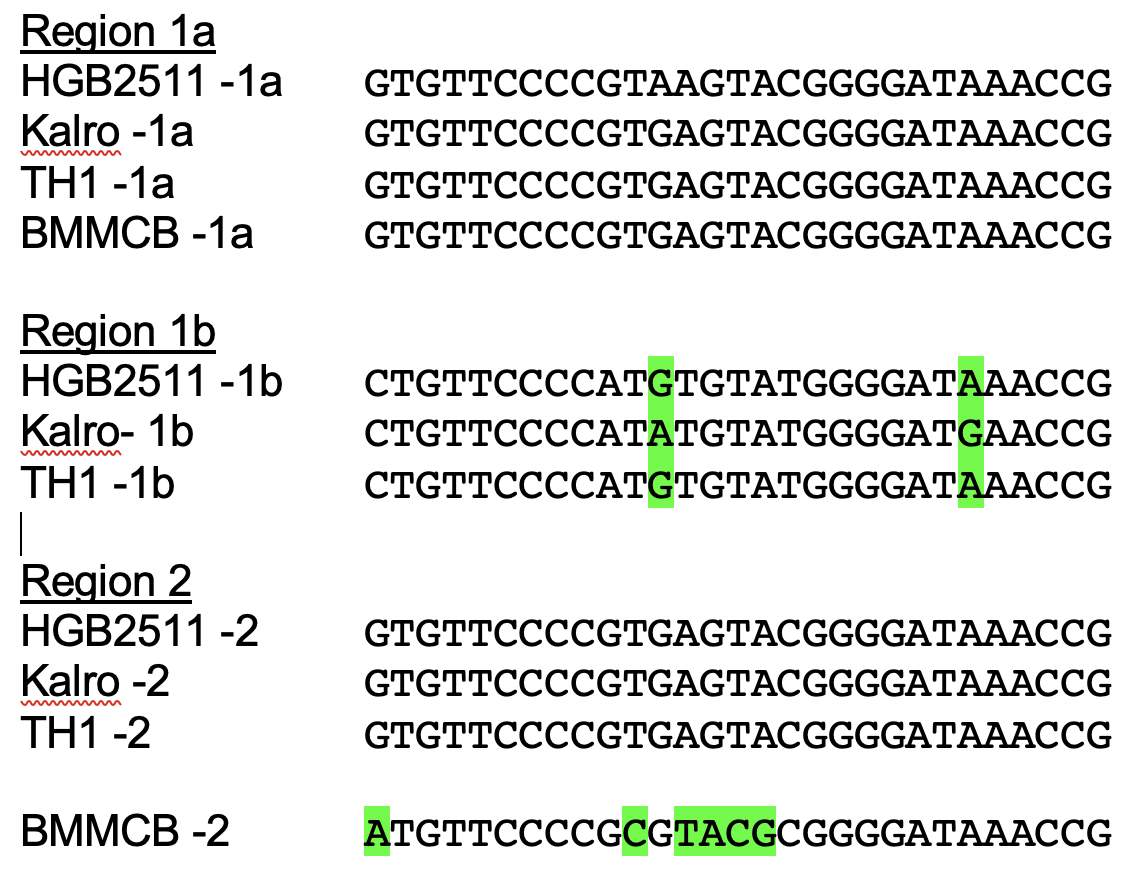


A manual search within the 9 selected genomes (HGB2511, ID10, xg97, Kalro, XN45, VH1, and TH1) for CRISPR repeats was conducted. Blastn (query coverage >0% and identity >0%) was used to search for sequence similarity to the previously published *Xenorhabdus* consensus CRISPR repeat [1]. This search yielded the same results as CRISPRdetect with two exceptions: the manual search did not detect the BMMCB region 2 repeats found by CRISPRdetect, but it did reveal five potential CRISPR repeat regions in ID10 that were not found by CRISPRdetect. Two of these were in region 1a and are denoted 1ai and 1aii, two were in region 1b, denoted 1bi and 1bii, and one was in region 2. Each is predicted to encode at most one spacer. To further analyze the ID10 repeat regions, the sequences were aligned manually with the CRISPRdetect consensus repeat sequence from each region, shown below highlighted in blue, with a 32-bp spacer region indicated by “X”. In addition, the last repeat-spacer-repeat sequence of each region in HGB2511 was included for comparison, since these were called by CRISPRdetect. Differences with the consensus are noted with yellow highlighting.


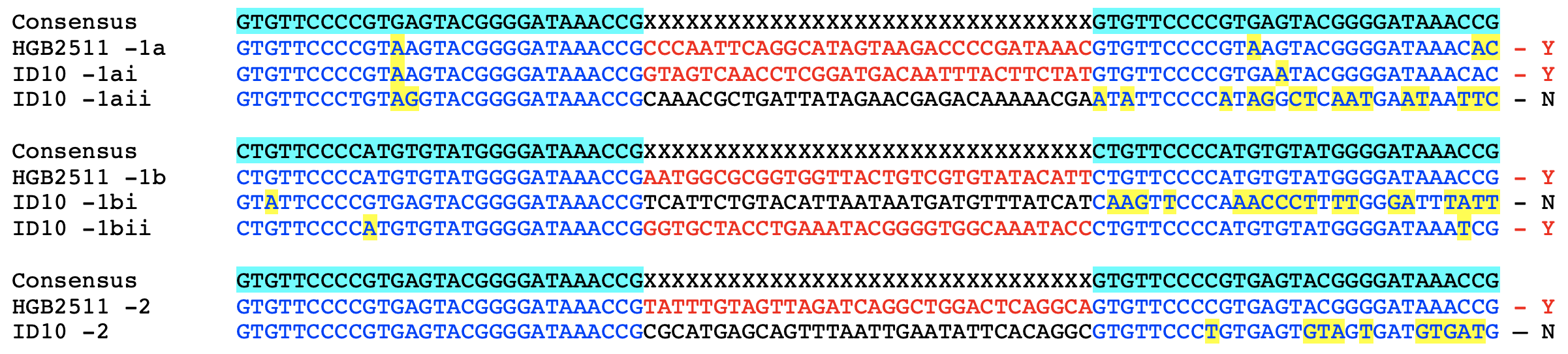


Since ID10 1ai and ID10 1bii regions have the same or fewer differences from consensus as the last HGB2511 repeat for each region, we have included these as bona fide, single spacer CRISPR loci, with the spacer sequence highlighted in red. Consistent with this call, these CRISPR repeats and spacer are syntenic with the longer CRISPR arrays found in HGB2511, the Kenyan clade strains, and TH1 (See Fig. 8 in the main text). However, the ID10 1aii, ID10 1bi, and region 2 have numerous differences from consensus in the second repeat and were not considered likely to encode CRISPR RNA.

**Protospacers and self-targeting immunity**

Four of the spacers from the *Xenorhabdus* strains tested have self-targeting protospacers: Kenyan clade spacer 1b-1 has a self-targeting protospacer in a *palA/fhaB* gene (e.g., Kalro QL128_RS09640, coordinates 2129819-2143876); Kenyan clade spacer 1b-3 has a self-targeting protospacer in an ABC transporter-related protein (e.g., Kalro QL128_RS13080, coordinates 2951989-2952618); TH1 spacer 1a-5 has a self-targeting protospacer in *folD* (XTH1_v2_2708, coordinates 2959194-2960069); and BMMCB spacer 1a-3 has self-targeting protospacers in homologs present in two predicted phage regions (LDNM01_v1_420008, coordinates 2447478-2449751 and LDNM01_v1_430003, coordinates 2456034-2458304).

We used a “guilt by association” approach to identify potential self-targeting immunity genes [2]. Using the MaGe comparative genome platform, we searched the genomic regions nearby protospacer genes for small ORFs that are present in self-targeting genomes but are absent in the other analyzed genomes.

*palA/fhaB*

In the Kenyan clade genomes, but not in ID10, HGB2511, or BMMCB, we found a cluster of four small ORFs encoded adjacent to the protospacer-containing *palA/fhaB* gene (QL128_RS09640) (See Fig. 8c in main text). These are predicted to encode a predicted *fhaB* fragment (QL128_RS09660), a HTH cro/C1-type domain-containing protein (QL128_RS09655), a *symE* toxin homolog (QL128_RS09650), and a DUF2247 domain-containing protein (QL128_RS09645). The DUF2247 gene fulfills the criteria to be an Acr candidate, since it is less than 200 aa (171aa), is encoded in the same orientation and downstream of the protospacer-encoding gene and is within four ORFs of an HTH domain-containing gene (QL128_RS09655) that is predicted to function as an “Aca” transcriptional regulator [2]. DUF2247 is also known as “imm38” and is found in poly-immunity loci [3].


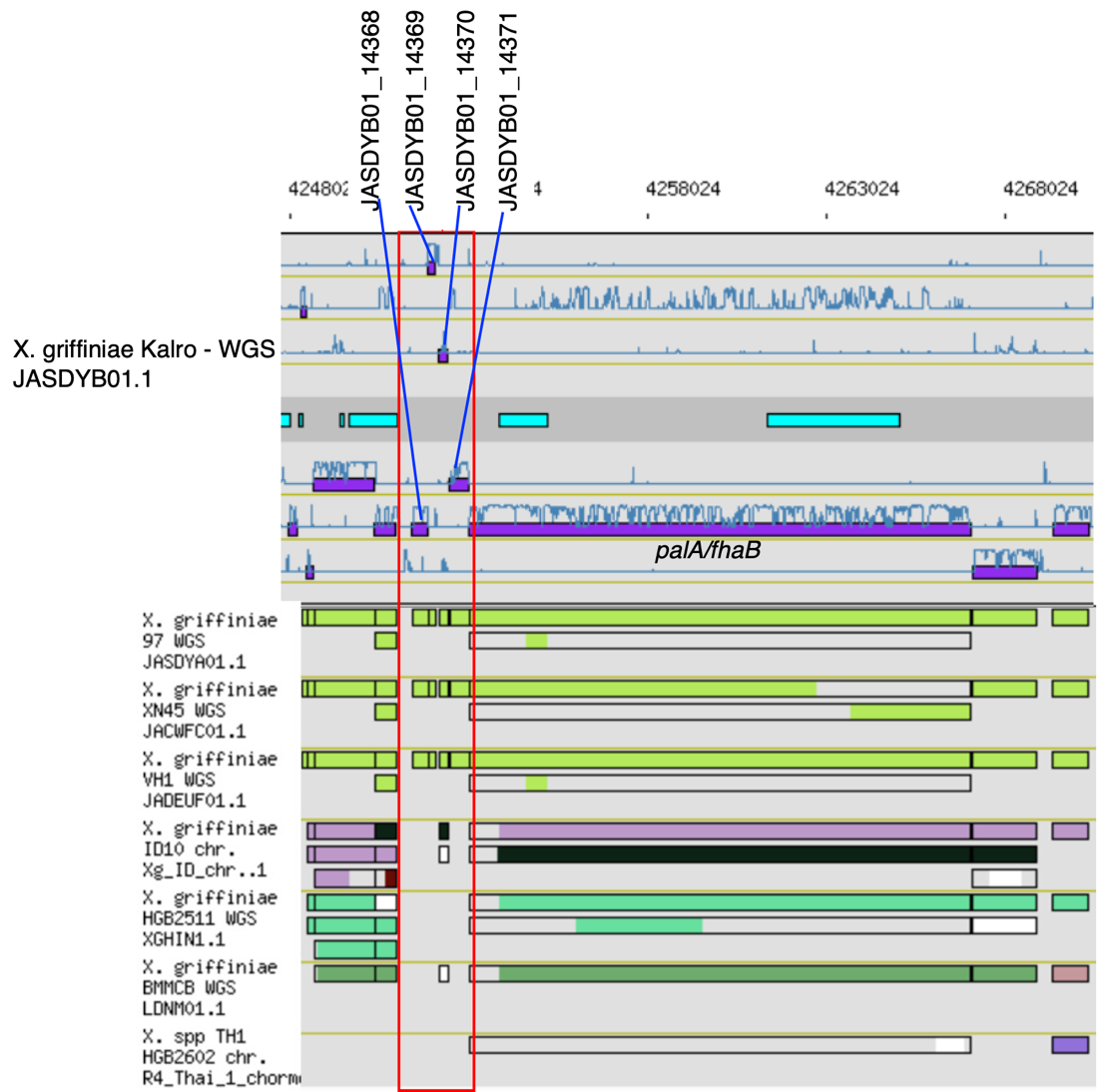


*ABC transporter-related protein*

For the Kenyan subclade spacer 1b-3, no clear evidence for Aca/Acr genes was observed in the genomic region surrounding the protospacer-containing genes (e.g., QL128_RS13080), predicted to encode ABC transporter-related proteins. Small open reading frames (e.g., QL128_RS13100, 83aa; QL128_13090, 51 aa; QL128_13065, 46 aa; QL128_13065, 59 aa; QL128_RS13050: 113aa, cupin domain containing) were present 3-5 Kb away from the protospacer-containing gene, but none was predicted to include an HTH-domain indicative of an Aca.


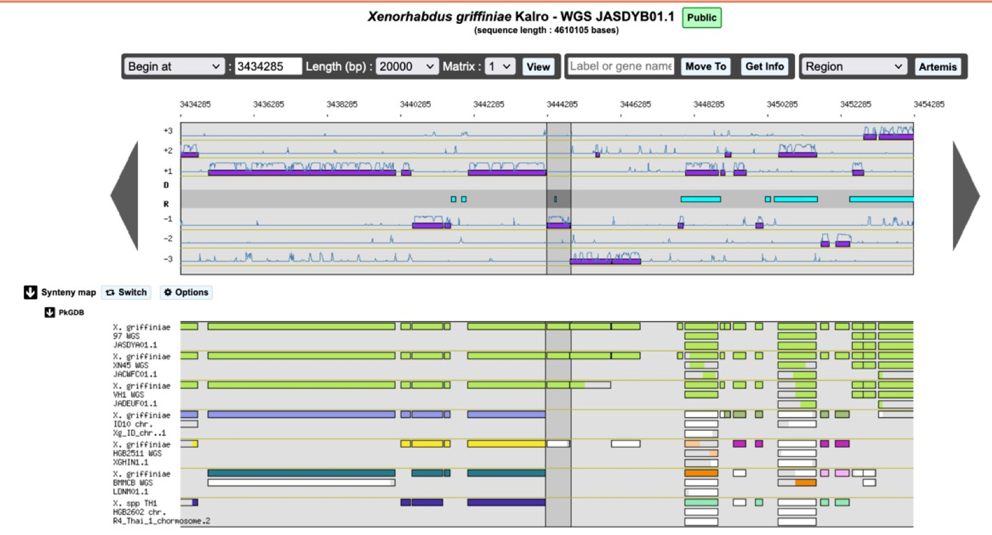


*folD*

In the TH1 genome, but not the other analyzed genomes, we found three small ORFs encoded in the vicinity of the *folD* protospacer-containing gene (bright green block arrow) with identity to TH1 spacer 1a-5. One of these, XTH1_v2_2706, is predicted to encode an integrase (black block arrow), while the other two, XTH1_v2_2705 and _2707 (pink block arrows) are predicted to encode proteins of unknown function of 38 aa and 71 aa, respectively. Neither is annotated as having an HTH-domain. Both HGB2511 and TH1 *folD* were flanked by *arg* tRNA and a S22 rRNA loci, but upstream of the *arg* tRNA they diverged. HGB2511 had phage-related genes (orange block arrows) and TH1 had a large locus (beginning of which is shown as blue lined block arrow) predicted to encode a non-ribosomal peptide synthetase.


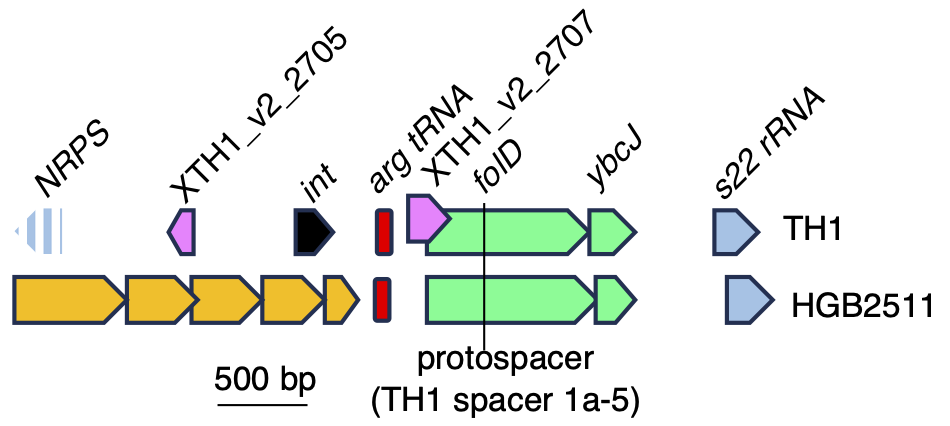


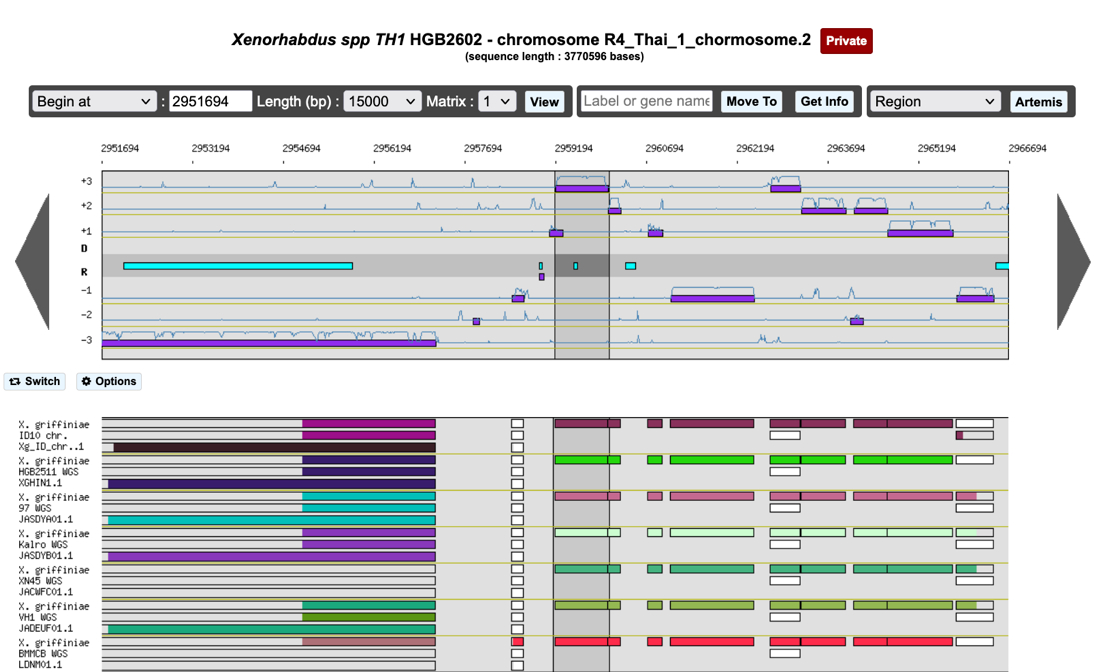


**References**

1. Veesenmeyer JL, Andersen AW, Lu X, Hussa EA, Murfin KE, Chaston JM, et al. NilD CRISPR RNA contributes to *Xenorhabdus nematophila* colonization of symbiotic host nematodes. Mol Microbiol. 2014;93:1026–42.

2. Makarova KS, Wolf YI, Koonin EV. In silico approaches for prediction of anti-CRISPR proteins. J Molec Biol. 2023;435:168036.

3. Zhang D, de Souza RF, Anantharaman V, Iyer LM, Aravind L. Polymorphic toxin systems: Comprehensive characterization of trafficking modes, processing, mechanisms of action, immunity and ecology using comparative genomics. Biol Direct. 2012;7:18.
